# Switchable Bandpass/Bandstop Filter Using Liquid Metal Alloy as Fluidic Switch

**DOI:** 10.3390/s19051081

**Published:** 2019-03-03

**Authors:** Eiyong Park, Minjae Lee, Sungjoon Lim

**Affiliations:** School of Electrical and Electronics Engineering, College of Engineering, Chung-Ang University, 84 Heukseok-ro, Dongjak-gu, Seoul 06974, Korea; rntqkdl9@naver.com (E.P.); iamlmj720@gmail.com (M.L.)

**Keywords:** bandpass filter (BPF), bandstop filter (BSF), liquid metal, micro-pump, switchable

## Abstract

In this paper, we propose a switchable band-pass/band-stop filter using liquid metal alloy as a fluidic switch. The filter is designed based on the Chebyshev response and implemented using a three-stage quarter-wavelength resonant structure. The fluidic switch is realized by injecting eutectic gallium–indium (EGaIn) in the microfluidic stubs, engraved in the polydimethylsiloxane (PDMS) material. When the fluidic switch selects the short stub using a micro-pump and microprocessor for switching, the filter acts as a bandpass filter (BPF) with the short stubs. When the fluidic switch selects the open stub, the filter acts as the bandstop filter (BSF) with the open stubs. At the BPF mode, the center frequency is 2.5 GHz and the 1-dB bandwidth is 1.75–3.07 GHz. The insertion loss is 0.5-dB ± 0.4-dB. At the BSF mode, the 15-dB bandstop bandwidth is 2.4–2.65 GHz with 2.5 GHz center frequency.

## 1. Introduction

A switchable bandpass/bandstop filter has received particular attention as it enables radio systems to be operated in an interference-congested environment [1], reduces the size [2], solves the complexity problem [3], and reduces the cost [4,5,6,7]. For a dynamic interference situation, the bandpass/bandstop filters (BPF/BSF) are used in the bandstop mode to reduce the high-power interference close to the desired signal, while the bandpass filter is selected for a lower interference environment [8].

Filters occupy a large space as compared to other typical radio components, and the filters are required in modern wireless systems [8]. Therefore, the higher-order filter [9] wideband with a smaller footprint has been required. Recently, tunable filters with bandpass/bandstop using new structures such as electromagnetic bandgap (EBG), closed-ring resonator, and two- and four-pole resonators [2,10] have been investigated. In these filters, active devices such as a varactor diode, pin diode, radio frequency micro-electro-mechanical system (RF MEMS), or some other actuation mechanisms have also been employed to facilitate the switching. The proposed structure consists of a quarter-wavelength structure and a liquid-metal that contains both liquid and metal properties. Compared to electrical or mechanical switches, a fluidic switch provides several advantages such as a simplified structure, wide tuning range, low loss at a high frequency, high power handling capability, cost-effectiveness, the lack of need for biasing circuits, and no interference or scattering problems created by biasing circuits [11,12,13,14,15,16,17]. On the other hands, a fluidic switch has limitations of switching speed and reliability. Nevertheless, to investigate the possibility of fluidical tunability in an RF regime, several RF components integrated with microfluidic channels have been investigated in which liquid metal is used for tuning, such as sensors [18], reconfigurable antennas [19,20], amplifiers [21], resonators [22], filters [23,24,25], and baluns [26]. 

In this paper, the switchable filter is designed, fabricated, and measured. We utilized a microfluidic channel to switch BPF and BSF using the fundamental third-order quarter-wavelength structure to operate at the 2.5 GHz band to ensure its wide utilization in wireless communication systems. The fluidic switches are loaded between the 50-ohm transmission line and the short/open stubs to select either the short or open stub. When the fluidic switch selects the short stub, the proposed filter is working as a BPF. When the fluidic switch selects the open stub, the proposed filter is working as a BSF. Consequently, the simulation and measurement results are highly consistent.

## 2. Switchable Bandpass/Bandstop Filter Design

**Figure 1:**
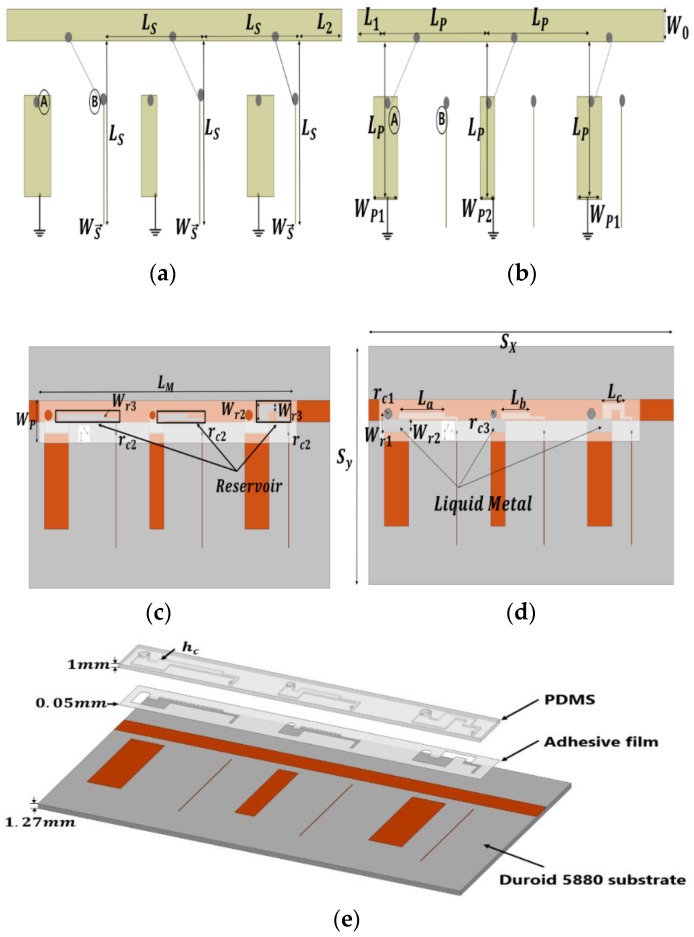
Schematic of the proposed switchable filter: (**a**) bandstop filter (BSF) as switching to B; (**b**) bandpass filter (BPF) as switching to A (L_s_ = 24 mm, L^p^ = 20 mm, L_1_ = L_2_ = 5 mm, W_0_ = 4 mm, W_s_ = 0.1 mm, W_p1_ = 6.3 mm, W_p2_ = 3.7 mm). Depicted is the top view of (**c**) BSF state (L_M_ = 68 mm, W_p_ = 8 mm, S_y_ = 45 mm, S_r_ = 79.4 mm, r_c1_ = 1 mm, r_c2_ = 0.25 mm, r_c3_ = 0.75 mm, L_a_ = 12 mm, L_b_ = 7.4 mm, L_c_ = 5.85 mm), (**d**) BPF state, and (**e**) a trimetric view (h_c_ = 0.5 mm) of the proposed filter.

Figure 1 shows the fundamental structure and principle of the switchable bandpass/bandstop filter. First, the filter is designed based on the 0.5 dB ripple, N = 3, and Chebyshev response, and is implemented by a quarter-wavelength resonant structure at a center frequency of 2.5 GHz. The length of each section is a quarter of the wavelength, λ_g_/4, except the port section. The width of the main line is matched with 50 Ω. The width of each stub is determined by the characteristic impedance.

In a BPF, the characteristic impedance (*Z*_0*n*_) is expressed by:

(1) $$Z_{0n}=\frac{\pi \times Z_{0}\times \Delta }{4\times g_{n}}$$

In a BSF, the characteristic impedance is expressed by:

(2) $$Z_{0n}=\frac{4\times Z_{0}}{\pi \times g_{n}\times \Delta }$$

where *g*_n_ is the 0.5 dB equal ripple coefficient. For N = 3, *g*_1_, *g*_2_, and *g*_3_ are 1.5956, 1.0967, and 1.5963, respectively. The fractional bandwidth (∆) is calculated as 1.27 to match the center frequency of the BPF with the BSF. 

Figure 1a,b shows the principle of the proposed filter. In order to achieve the switchable bandpass/bandstop filter, the switch is loaded between the 50-ohm transmission line and the short/open stub, as illustrated in Figure 1. For instance, in Figure 1a, when the switch selects B, the filter acts as a BSF because of the open stubs. On the contrary, when the switch selects A, the stubs of the filter become shorted, and the filter operates as a BPF, as shown in Figure 1b.

We apply this principle to our proposed filter to create the fluidic switch. When the fluidic switch selects B (the liquid metal is injected into the fluidic open stub using a micro-pump), the stub acts as shown in Figure 1a, and the part of the microfluidic channel acts as a reservoir for the liquid metal to minimize the effect of the reservoir, as shown in Figure 1c. Figure 1d shows the state of the BPF. When the fluidic switch selects A (liquid metal is moved in the fluidic short stub using micro-pump), the liquid metal connects the transmission line and the stub with the vias, and the stub acts as shown in Figure 1b. Figure 1e shows a birds-eye view of the proposed filter. The switchable bandpass-to-bandstop filter consisted of a copper plate, substrate, adhesive film, and the polydimethylsiloxane (PDMS). The substrate RT/duroid 5880 (Rogers, Killingly, CT, USA) with a thickness of 1.27 mm and dielectric constant of 2 was used to realize our proposed filter. The adhesive film was used for the bonding between the substrate and the PDMS layer. The adhesive film ARcare92561 (permittivity 3 and thickness 0.05 mm) was provided by Adhesives Research, Glen Rock, PA, USA. The PDMS (permittivity 3.2) was used to fabricate the microfluidic channel. 

**Figure 2:**
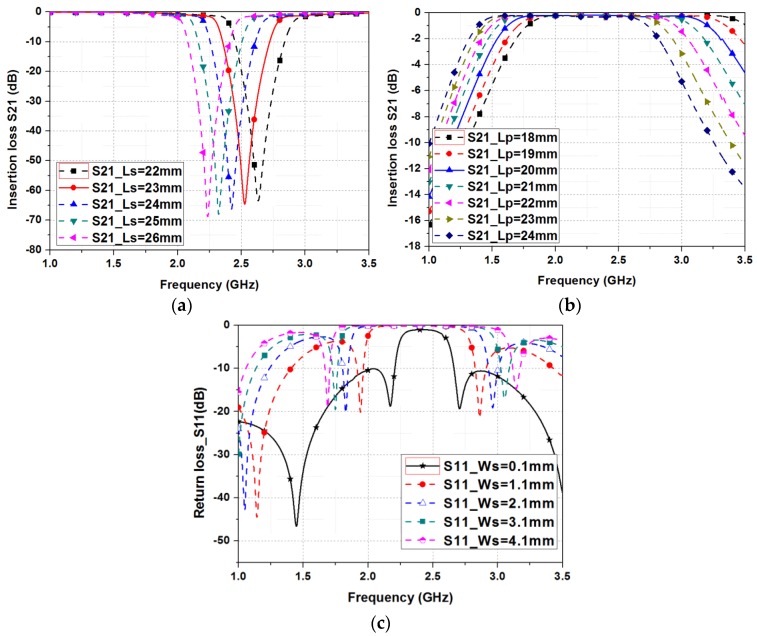
Simulated S_21_ of the proposed filter for: (**a**) different *L*_r_; (**b**) different *L_s_*; (**c**) different *L*_c_.

Figure 2 shows the simulated insertion losses (S_21_) of the proposed filter for different geometrical parameters. In the proposed filter, the quarter-wavelength (λ_g_/4) is 22.738 mm at the center frequency of 2.5 GHz. Figure 2a shows the insertion loss of the BSF when the liquid metal is injected in the open stub (the fluidic switch selects B) by varying the length of the open stub (L_s_) from 22–25 mm. As L_s_ increases, the resonant frequency is decreased. Figure 2b shows the insertion loss of the BPF when the liquid metal is moved in the short stub (the fluidic switch selects A) by varying the length of the short stub (L_p_) from 18–24 mm. As L_p_ increases, the bandpass frequency is decreased. Figure 2c shows the return loss of the BSF by varying the width of the open stub (W_s_) from 0.1–4.1 mm. As Ws decreases, the bandwidth is decreased, and the return loss is decreased. Finally, L_s_ = 23 mm, L_p_ = 20 mm and W_s_ = 0.1 mm are decided for 2.5 GHz band operation. 

**Figure 3:**
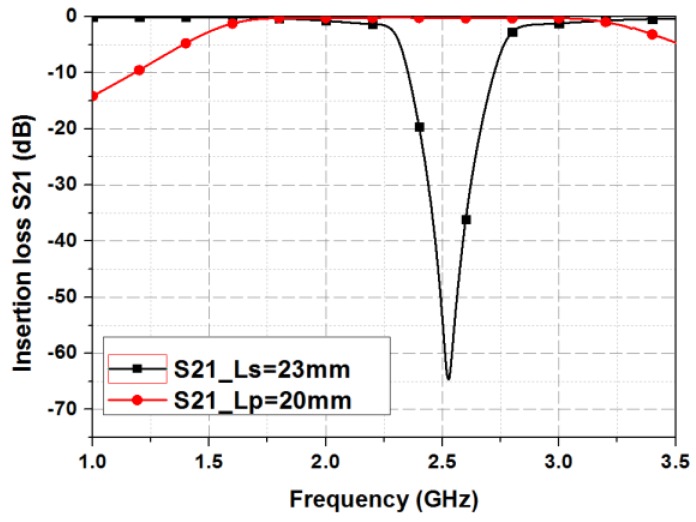
Simulated S-parameters of the proposed filter.

The simulation results of the final design are shown in Figure 3. At the BSF mode, the 15-dB bandstop bandwidth is 2.4–2.65 GHz at the center frequency of 2.5 GHz. At the BPF mode, the center frequency is 2.5 GHz, and the 1-dB bandwidth is 1.75 GHz–3.07 GHz. The insertion loss is 0.5-dB ± 0.4-dB in the 1-dB bandwidth with a return loss higher than 15-dB.

## 3. Fabrication and Measurement

In order to fabricate the proposed filter, Duroid 5880 was used as the substrate. The metallic pattern and truncated ground were designed on the copper plate. We used gold vias with radiuses of 0.25 mm. We used the PDMS material for a microfluidic channel because of its flexibility and durability [27]. It is also known that the surface does not respond to reagents [28]. The microfluidic channel was engraved in PDMS using a laser cutting machine and attached with the substrate using an adhesive film. The prototype sample was soldered with a 50-Ω subminiature-version-A (SMA) connector. The liquid metal used was eutectic gallium–indium (EGaIn).

**Figure 4:**
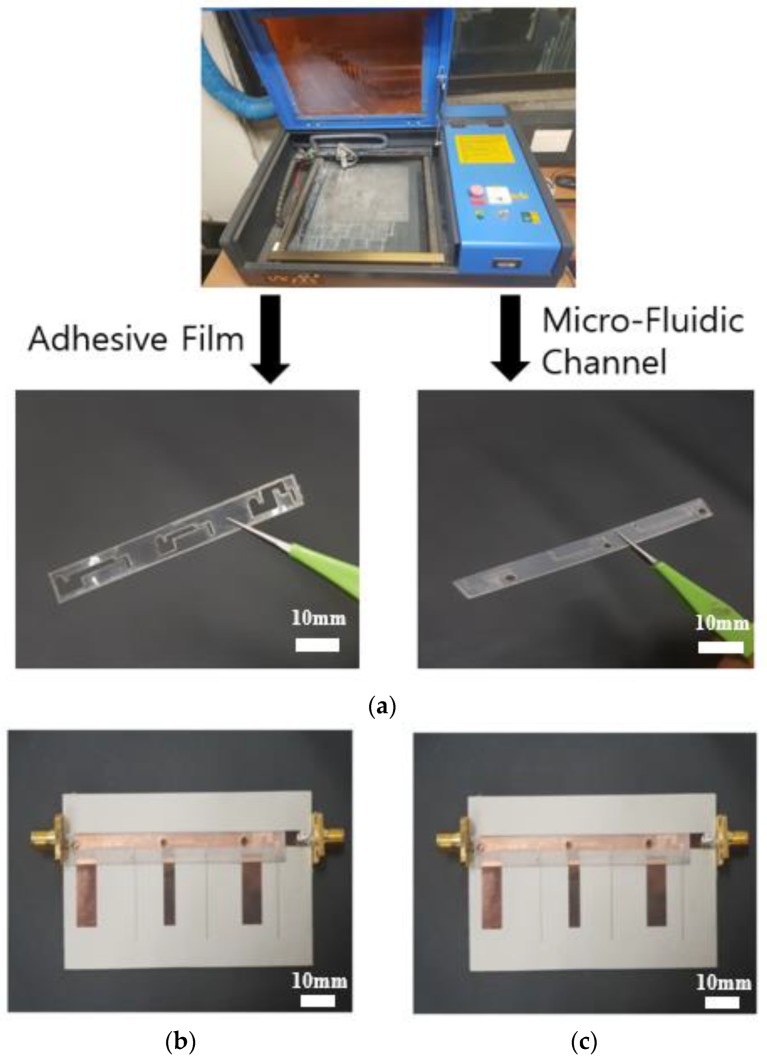
(**a**) The process of fabricating the fluidic channel and adhesive film using a laser cutting machine. The picture of the fabricated proposed filter (**b**) is shown without the microfluidic channel; (**c**) is shown with the added microfluidic channel.

Figure 4a shows the photograph of processing the microfluidic channel and adhesive film by using a laser machine. Figure 4b shows the picture of the fabricated proposed filter without the microfluidic channel. After making Figure 4a,b, the proposed filter is completed by combining Figure 4a,b, as shown in Figure 4c.

**Figure 5:**
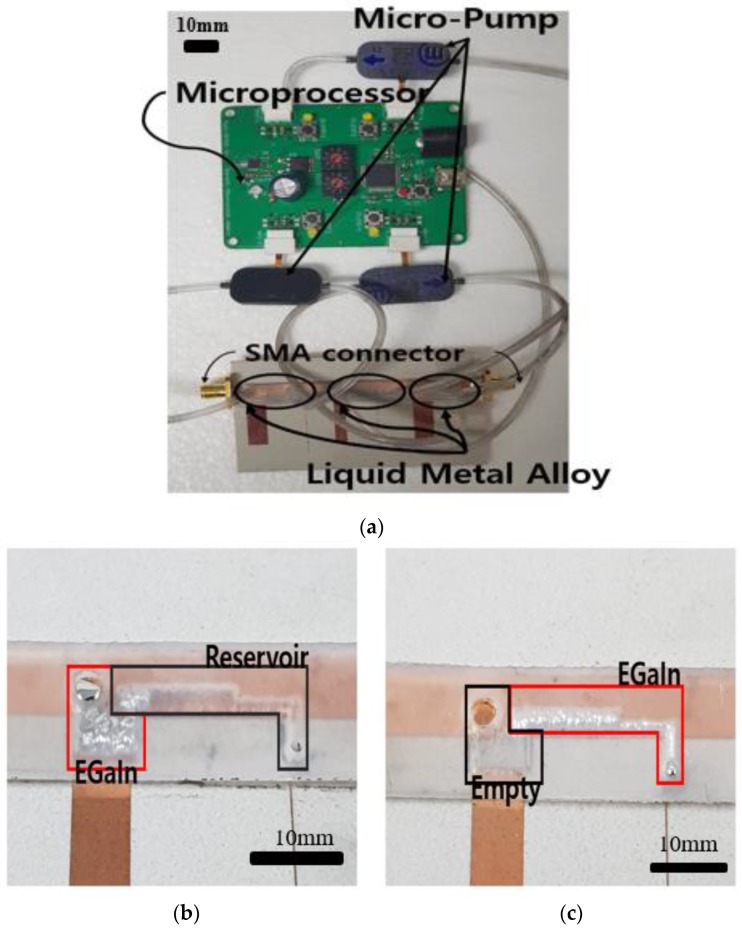
Photograph of (**a**) the fabricated sample of the proposed switchable bandpass-to-bandstop filter, (**b**) the fluidic switch where the liquid metal is in the reservoir, and (**c**) the fluidic switch when the channel is filled by liquid metal.

Figure 5a shows the photograph of the proposed filter sample. We used three mp-6 micro-pumps (Bartels Mikrotechnik GmbH, Germany) to transfer the liquid metal from the reservoir to the short stub or open stub by exerting the pressure provided through the tube and mp6-OEM controller microprocessor. The power consumption of each micro-pump was 50 mW. The instill rate of the micropump used in the proposed filter is 66.7–100 mm/s when the channel width is 0.51 mm [29]. Because the filter has a channel width of 2.5 mm and a length of 6.3 mm, the switching speed is estimated to be 0.3 ms. 

**Figure 6:**
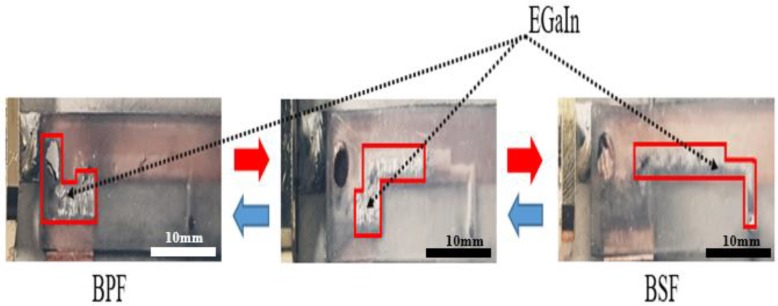
Snapshot pictures for the liquid metal moving direction.

Table 1 shows the characteristics of each parameter according to each tuning method. Figure 5b shows the state when the fluidic switch selects the short stub. In this case, the filter operates as a BPF. When the fluidic switch selects the open stub, the liquid metal is driven to the open stub, and the filter operates as a BSF, as shown in Figure 5c. Figure 5c shows the state after transferring the liquid metal to the short stub. Figure 6 shows how the EGaIn liquid metal is moving. The mp6-micropump is actuated by two piezoelectric disks which push the liquid metal to move [30]. When the liquid metal is injected, the proposed filter mode is switched to BSF from BPF. 

**Figure 7:**
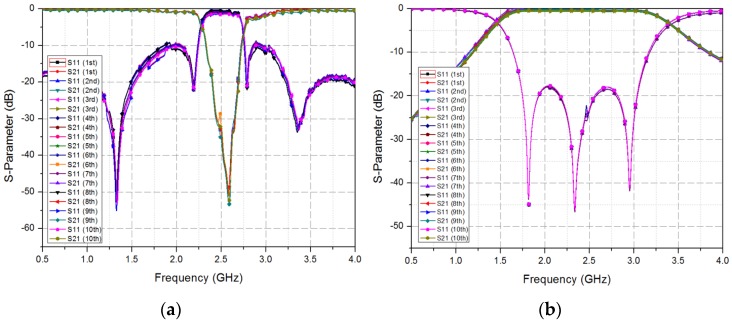
Measured S-parameter results of the fabricated filter after 10 times switching: (**a**) BSF and (**b**) BPF.

In this work, the fluidic switch is designed and fabricated after considering the oxidation problem, repeatability, and reversibility of the liquid metal. The liquid metal tends to form solid oxide shells on the surface when exposed to air/oxygen. The oxidation problem is solved using several proper surface treatments. For instance, hydrochloric acid (HCl) is used to prevent the formation of oxides. An EGaIn channel is pre-processed in Nafion and then rinsed in HCl solution. Nafion absorbs HCl and progressively releases it into steam to prevent oxide formation. In this work, we used EGaIn for a liquid metal because it does not suffer from an oxidation problem [31]. To repeatably inject and extract the liquid metal, the fluidic channel is designed with four different widths. From the turbulence and entrance effects, the step-down width of the fluidic channel can make the liquid metal flow smoothly [32,33]. The repeatability and reversibility of the proposed filter are tested by switching the fluidic switch 10 times. Figure 7 shows the measured S-parameter results of the BSF and BPF at each injection and extraction. Therefore, the oxidation problem does not affect the performance of the proposed filter, and the proposed filter can be repeatably used.

**Figure 8:**
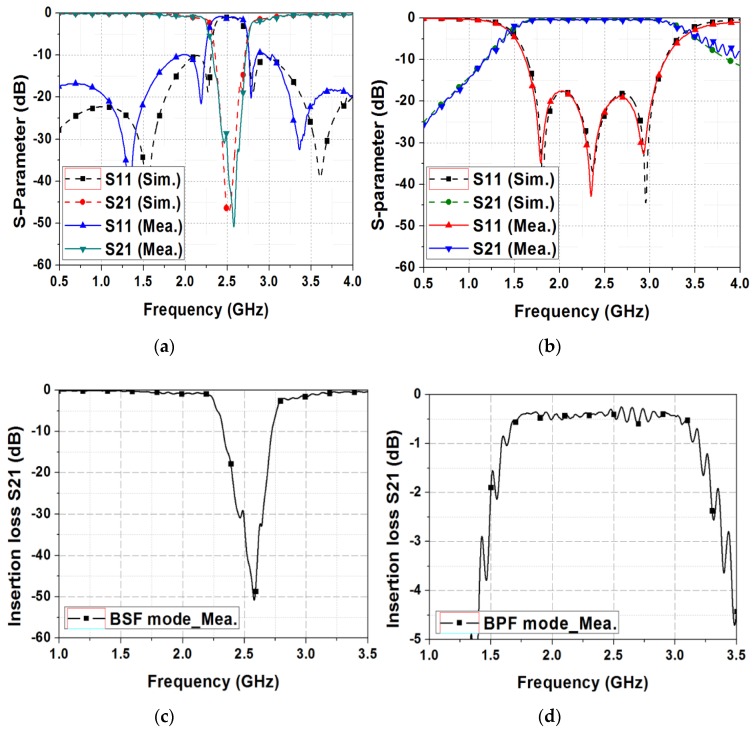
Simulation and measurement results when (**a**) the fluidic switch selects the open stub and (**b**) the fluidic switch selects the short stub. Measured insertion losses are depicted for (**c**) BSF and (**d**) BPF.

The S-parameter of the proposed filter is measured by an Anritsu MS2038C (Anritsu, Kanagawa, Japan) vector network analyzer. For full two-port calibration, SOLT (short circuit, open circuit, load and thru) calibration is performed using the TOSLKF50A-20 (Anritsu, Kanagawa, Japan) calibration kit. Figure 8 shows the measurement results of the proposed switchable bandpass to bandstop filter. The simulation and measurement results show excellent agreement when the fluidic switch selects the open stub or short stub, as shown in Figure 8a,b, respectively. A slight difference between simulation and measurement results in Figure 8a is observed because of the misalignment of microfluidic channels and the printed circuit boards. Figure 8c shows the measured insertion losses of the BSF when the fluidic switch selects the open stub. At the BSF mode, the 15-dB bandstop bandwidth is 2.4–2.65 GHz at the center frequency of 2.5 GHz. Figure 8d shows the measured insertion loss of the BPF when the fluidic switch selects the short stub. At the BPF mode, the center frequency is 2.5 GHz, and the 1-dB bandwidth is 1.75 GHz–3.07 GHz. The insertion loss is 0.5-dB ± 0.4-dB in the 1-dB bandwidth with a return loss higher than 15-dB.

## 4. Conclusions

We herein proposed a switchable bandpass/bandstop filter using liquid metal alloy as a fluidic switch. To switch the filter mode, the fluidic channel and liquid metal were used; they were driven by a micro-pump and microprocessor. When the fluidic switch selects the open stub, the proposed filter operates as the BSF. When the switch selects the short stub, the proposed filter operates as the BPF. At the BPF mode, the center frequency is 2.5 GHz and the 1-dB bandwidth is 1.75–3.07 GHz. The insertion loss is 0.5-dB ± 0.4-dB in the 1-dB bandwidth with a return loss better than 15-dB. At the BSF mode, the 15-dB bandstop bandwidth is 2.4–2.65 GHz with the center frequency of 2.5 GHz. The proposed switchable filter is numerically and experimentally demonstrated. The simulation and measurement results show excellent agreement.

## Figures and Tables

**Table 1:** Parameters of other tuning techniques [3].

	Liquid Metal (Proposed Work)	YIG	BST	Schottky Diode	Pin Diode	MEMS
Tuning speed	Millisecond	Millisecond	Nanosecond	Nanosecond	Nanosecond	Microsecond
Operating frequency	unlimited	limited	limited	limited	limited	limited
Temperature Sensitivity	High	High	High	Low	Low	Low
Biasing	No needed	Magnetic Field	Electric Field	Electric Field	Electric Field	Electric Field
Power consumption	50 mW	0.5–5 W	0	0	20–30 mA	0
Cost	Low	High	High	Low	Low	High
